# Intravoxel Incoherent Motion Metrics as Potential Biomarkers for Survival in Glioblastoma

**DOI:** 10.1371/journal.pone.0158887

**Published:** 2016-07-07

**Authors:** Josep Puig, Javier Sánchez-González, Gerard Blasco, Pepus Daunis-i-Estadella, Christian Federau, Ángel Alberich-Bayarri, Carles Biarnes, Kambiz Nael, Marco Essig, Rajan Jain, Max Wintermark, Salvador Pedraza

**Affiliations:** 1 Imaging Research Unit, Institut de Diagnostic per la Imatge (IDI), Girona Biomedical Research Institute (IDIBGI), Hospital Universitari Dr Josep Trueta, Girona, Spain; 2 Philips Healthcare Ibérica, Madrid, Spain; 3 Department of Computer Science, Applied Mathematics, and Statistics, University of Girona, Girona, Spain; 4 Department of Diagnostic and Interventional Radiology, Centre Hospitalier Universitaire Vaudois (CHUV) and University of Lausanne, Lausanne, Switzerland; 5 Department of Radiology, Neuroradiology Division, Stanford University, Palo Alto, United States of America; 6 Biomedical Imaging Research Group (GIBI2^30^), La Fe Polytechnics and University Hospital, La Fe Health Research Institute, Valencia, Spain; 7 Department of Radiology, Icahn School of Medicine at Mount Sinai, New York, United States of America; 8 Department of Radiology, University of Manitoba, Winnipeg, Canada; 9 Division of Neuroradiology, Department of Radiology, NYU Langone Medical Center, New York, United States of America; Instituto de Investigación Sanitaria INCLIVA, SPAIN

## Abstract

**Objective:**

Intravoxel incoherent motion (IVIM) is an MRI technique with potential applications in measuring brain tumor perfusion, but its clinical impact remains to be determined. We assessed the usefulness of IVIM-metrics in predicting survival in newly diagnosed glioblastoma.

**Methods:**

Fifteen patients with glioblastoma underwent MRI including spin-echo echo-planar DWI using 13 b-values ranging from 0 to 1000 s/mm^2^. Parametric maps for diffusion coefficient (*D*), pseudodiffusion coefficient (D*), and perfusion fraction (*f*) were generated for contrast-enhancing regions (CER) and non-enhancing regions (NCER). Regions of interest were manually drawn in regions of maximum *f* and on the corresponding dynamic susceptibility contrast images. Prognostic factors were evaluated by Kaplan-Meier survival and Cox proportional hazards analyses.

**Results:**

We found that *f*_CER_ and D*_CER_ correlated with rCBF_CER._ The best cutoffs for 6-month survival were *f*_CER_>9.86% and D*_CER_>21.712 x10^−3^mm^2^/s (100% sensitivity, 71.4% specificity, 100% and 80% positive predictive values, and 80% and 100% negative predictive values; AUC:0.893 and 0.857, respectively). Treatment yielded the highest hazard ratio (5.484; 95% CI: 1.162–25.88; AUC: 0.723; *P* = 0.031); *f*_CER_ combined with treatment predicted survival with 100% accuracy.

**Conclusions:**

The IVIM-metrics *f*_CER_ and D*_CER_ are promising biomarkers of 6-month survival in newly diagnosed glioblastoma.

## Introduction

High-grade neoplasms produce a complex microvascular network to satisfy a growing need for nutriments and oxygen [1], and glioblastoma is among the most angiogenic tumors [2]. Cerebral blood volume (CBV) correlates with the degree of angiogenesis and increased local perfusion correlates with tumor grading and survival [3–5]. Therefore, hemodynamic parameters influenced by vessel density and perfusion of the microvasculature, such as CBV and cerebral blood flow (CBF), can be used as surrogate biomarkers [6–8]. As these perfusion parameters can be measured by dynamic susceptibility contrast (DSC) MRI, this technique plays an important role in the baseline evaluation and follow-up of brain tumors. Recently, intravoxel incoherent motion (IVIM) has been proposed as an alternative perfusion MRI technique [9–18]. IVIM uses endogenous tracers to obtain perfusion-related indexes from diffusion-weighted imaging (DWI) datasets without contrast agents.

Considering the vascular bed as a random network of vessels where blood flows freely, Le Bihan et al. [19] demonstrated that IVIM could distinguish between water diffusion and the microcirculation of blood in the capillary network. In biological tissues, diffusion and perfusion are physically different phenomena, and the incoherent motion of spins, which can be understood as the spatial “mixing” of spins during the time of measurement in each image voxel, bi-exponentially reduces the signal amplitude observed when different diffusion b-values are applied [20]. In other words, DWI is also sensitive to perfusion because the flow of blood in randomly oriented capillaries mimics a diffusion process. IVIM modeling allows the extraction of two diffusion coefficients, one related to molecular diffusion restriction, called the diffusion coefficient (*D*), and another related to movements of blood in the microvasculature, called the pseudodiffusion coefficient (D*). A third parameter, the perfusion fraction (*f*), describes the fraction of incoherent signal arising from the vascular compartment in each voxel. In recent years, advances in MR hardware have allowed short-time acquisitions with multiple b-values and sufficient signal-to-noise ratio, reviving interest in IVIM for imaging tumors in the brain [8–12] and in body tissues where vascularity is important [18,21–25] for characterizing tumors and predicting or monitoring the response to treatment [9,12,26,27]. Recent evidence suggests that *f* values can help differentiate between low- and high-grade gliomas [10–12], and *f* correlates moderately with DSC rCBV [11]. In addition, in rodent models of glioma, *f* correlates positively with vessel density at histology [28].

To our knowledge, no data about the usefulness of IVIM-metrics to predict survival in glioblastoma patients are available. Therefore, we determined whether IVIM-metrics *D*, D*, and *f* are useful in predicting tumor response to treatment and survival in newly diagnosed glioblastoma, by analyzing them in contrast-enhancing regions (CER) and non-enhancing regions (NCER) surrounding the CER.

## Materials and Methods

### Patient characteristics

The ethics committee at Hospital Dr Josep Trueta approved this prospective study, and all patients provided written informed consent to participate in it. From November 2009 to March 2011, we enrolled 23 consecutive patients with newly diagnosed glioblastoma suspected on CT or MRI. After imaging, all lesions were biopsied. Eight (34.78%) patients were excluded from the study: five because histology ruled out glioblastoma and three because motion artifacts hindered image evaluation. Therefore, 15 patients (7 women; mean age, 66±11 years) were included. Patients were managed according to published guidelines [29]. The combination of surgery, radiotherapy, and chemotherapy with concomitant and adjuvant temozolomide was considered standard treatment. Patients did not receive corticosteroids before MRI. Survival was measured from the pretreatment MRI study to death.

### Conventional MRI

MRI was performed on a 1.5-T MR scanner (Gyroscan Intera 1.5T Master; Philips Healthcare, Best, the Netherlands) using an eight-channel head coil. Before contrast administration, we acquired axial T1WI SE (TR536ms, TE15ms), axial T2WI fast SE (TR4400ms, TE110ms), and axial FLAIR (TR8000ms, TE115ms, TI2200ms) sequences. We used a 230-mm field of view, 5-mm section thickness, and 256x192 matrix for these sequences. Five minutes after gadobutrol injection, we obtained axial T1WI SE (TR600ms, TE10ms) images parallel to the bicommissural line.

### IVIM MRI

24 axial DW images (TR3000/TE76ms) were obtained using single-shot spin-echo echo-planar imaging (EPI) before contrast-enhanced MRI. The EPI factor was 41, and the sensitivity-encoding factor was two. We used a 200-mm field of view, 5-mm section thickness, and 96 x 77 matrix. The measured pixel size was 2.4x2.9x5mm. We used 13 b-values: 0, 10, 20, 30, 50, 100, 150, 200, 350, 500, 650, 800, and 1000 sec/mm^2^. The total acquisition time was 3 minutes 48 seconds per patient.

### DSC-MRI

Using the same section orientations used for DW images, we acquired dynamic T2*-weighted gradient-echo echo-planar images (TR1800ms; TE25ms) during the first pass of a standard dose (0.1 mmol/kg) bolus of gadobutrol injected via an antecubital vein at 5 ml/s followed by 30 ml saline solution. To assure that steady-state magnetization was reached, a five series of dummy scans (i.e., the pulse sequence is run, but data are not acquired) were inserted immediately before the start of each perfusion series consisted of 50 dynamic acquisitions. Between 8 and 10 per-Gd baseline images were acquired. Based on T2WI and FLAIR images, we selected seven to ten sections through the tumor for PWI in a single TR with an in-plane resolution of 1.95x1.95 mm^2^ and slice thickness of 7 mm. The methods used for acquiring data and the algorithm for calculating CBV-corrected maps for contrast agent extravasation are described elsewhere [7].

### IVIM Image Processing

The IVIM model considers that two compartments exist in biologic tissue: a slow-moving compartment, where particles diffuse in a Brownian fashion as a consequence of thermal energy, and a fast-moving compartment (the vascular compartment), where water molecules moves as a consequence of forced blood circulation [19]. In the vascular compartment, D* describes the displacement of blood on a macroscopic level in an assumed randomly laid vascular network. For the perfusion to be physiologically meaningful, D* must be greater than D. Therefore:

$$S_{b}=S_{0}\left[fe^{-bD^{*}}+\left(1-f\right)e^{-bD}\right],$$ (1)

where *f* is the fraction of the diffusion linked to microcirculation (perfusion fraction), *D* is the diffusion parameter representing pure molecular diffusion (diffusion coefficient), and D* is the diffusion-related incoherent microcirculation; *S*_0_ is the signal intensity at a b-value of zero (i.e., without diffusion weighting), and *S*_*b*_ is the signal intensity for each b-value (i.e., at each diffusion gradient).

### Regions of Interest

A neuroradiologist with 20 years’ experience (S.P.) reviewed the anatomical images, using Olea Sphere V.2.0 software (Olea Medical, La Ciotat, France). NCER was defined as the hyperintense area surrounding the CER on FLAIR [30]. A fully automated deconvolution analysis was performed to create parametric images of CBV and CBF [31] in the MR Extended Workspace (Philips Healthcare, Best, the Netherlands). Due to the technique’s limitations in obtaining absolute CBF values, an extra ROI was placed in healthy gray matter as a reference [32]. To scale all CBF values, gray matter flow was established at the same level (65ml/100g/min) for all patients [33]. DW data were registered to the image with b-value = 0 s/mm^2^ using an affine transformation and a mutual information algorithm to avoid image distortion due to eddy currents. Images were analyzed with a computer program developed within the research group on the Philips Research Imaging Development Environment research platform using Interactive Data Language 6.3 (Research Systems Inc.; Boulder, CO, USA); this program fits every pixel to the three parameters in the model described in eq 1 using a Levenberg-Marquardt least-squares minimization algorithm [34]. The software generates three IVIM maps (one for *D*, one for D*, and one for *f*) in about 3 minutes. A reader (G.B. with 11 years’ experience) manually placed ROIs in the CER and NCER for each tumor, with maximal *f* on three contiguous axial sections. Mean ROI size was 32±12 mm^2^. Large vessels and cystic or necrotic tumor areas were excluded. The corresponding ROIs were then drawn on the DSC images (mean size, 30±14mm^2^) and the results were averaged for CER and NCER. To enable intraobserver reliabilities to be calculate, all measurements were repeated 1 month after the first determination. The observer was blinded to the clinical and outcome data of the patients.

### Statistical Analysis

Means and standard deviations were calculated for all parameters. Data were evaluated through the significance of the Pearson product-moment correlation coefficient. Linear regression analysis was performed using rCBV and rCBF values from DSC-MRI and the *f*, *D*, and D* values from IVIM. Receiver operating characteristic analysis was used to determine the optimal perfusion MRI parameter cutoffs for predicting 6-month survival. Prognostic factors included age, sex, Karnofsky Performance Score, treatment, volume of CER, volume of NCER, IVIM-metrics, and DSC-MRI parameters. Survival curves were calculated using the Kaplan-Meier method. We used the multivariate Cox proportional hazards model to adjust for the influence of prognostic factors. We used intraclass correlation coefficients (ICC) to compare measurements of rCBV, rCBF, *f*, *D*, and D* in CER and NCER, classifying intraobserver reliability as fair (ICC = 0.5–0.7), good (0.7–0.9), or almost perfect (>0.90). We also analyzed the variability of the measurements by Bland-Altman plots showing the mean difference between two methods of measurement, and 95% limits of agreement as the mean difference [35]. Minitab version 16.2.1 was used for statistical analyses (Minitab Inc.;State College,PA,USA). Significance was set at *P*<0.05.

## Results

### Patient Data

Table 1 summarizes patients’ clinical and imaging characteristics. All 15 patients died during the observation period. Survival was 10.6±6.23 months (range, 5–21 m) in patients receiving standard treatment and 4.7±2.81 months (range,1–8.5 m) in patients not receiving standard treatment.

**Table 1:** Patient characteristics.

Characteristic	Datum^a^
**Male:Female**	8:7
**Age**	66±11 (42–79)
**Motor deficit (%)**	46.67
**Language deficit (%)**	33.33
**Karnofsky Performance Score**	89.33±9.61(70–100)
**Volume of CER (mL)**	18.01±11.91 (7.34–49.85)
**Volume of Necrosis (mL)**	6.60±5.77 (0.59–21.21)
**Volume of NCER (mL)**	48.65±26.38 (16.45–119.23)
***f***_**CER**_ **(%)**	10.80±2.49 (7.28–15.12)
***D***_**CER**_ **(x10**^**-3**^**mm**^**2**^**/s)**	1.064±0.165 (0.804–1.378)
D*_CER_ **(x10**^**-3**^**mm**^**2**^**/s)**	24.665±5.140 (16.802–33.163)
**ADC**_**CER**_**(x10**^**-3**^**mm**^**2**^**/s)**	110.78±18.13 (84.82–142.89)
**rCBF**_**CER**_ **(ml/100g/min)**	51.27±21.56 (18.69–86.91)
**rCBV**_**CER**_ **(ml/100g)**	4.69±1.59 (2.27–7.37)
**max rCBF**_**CER**_ **(ml/100g/min)**	151.63±21.01 (112–181.84)
**max rCBV**_**CER**_ **(ml/100g)**	12.73±3.31 (8.67–19.07)
***f***_**NCER**_ **(%)**	2.34±0.99 (1.02–3.83)
***D***_**NCER**_ **(x10**^**3**^**mm**^**2**^**/s)**	1.488±0.270 (1.097–1.976)
D*_NCER_ **(x10**^**3**^**mm**^**2**^**/s)**	4.632±2.264 (1.133–8.614)
**rCBF**_**NCER**_ **(ml/100g/min)**	23.65±10.71 (11.03–46.55)
**rCBV**_**NCER**_ **(ml/100g)**	1.47±0.76 (0.67–3.61)
**Treatment (n)**	
**Standard**	5
**Non-standard (surgery only)**	8
**Non-standard (palliative care)**	2
**Survival (months)**	6.7±4.83 (1–21)
**Standard treatment**	10.6±6.23 (5–21)
**Non-standard (surgery only)**	4.81±2.53 (1–8.5)
**Non-standard (palliative care)**	4.5±3.54 (2–7)

^a^Unless otherwise specified, data are means ± standard deviations, with ranges in parentheses.

### Associations and correlations between DSC-MRI parameters and IVIM-metrics

**Fig 1:**
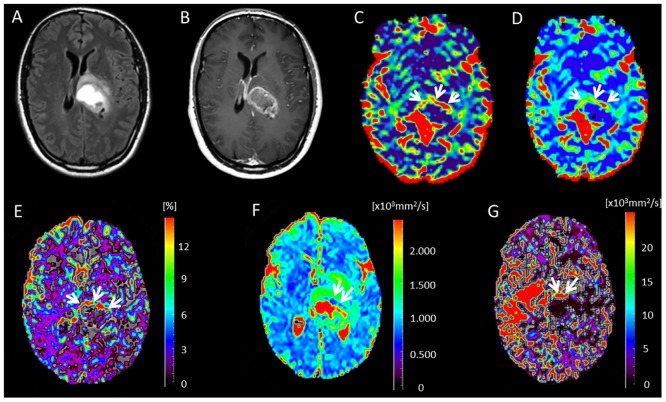
Glioblastoma in a 62-year-old woman. (A) Axial FLAIR image. (B) Contrast-enhanced T1-weighted image shows a rim-enhancing tumor. (C) Cerebral blood flow map obtained with DSC-MRI shows hyperperfusion signals predominantly in the left margin of the tumor (*arrows*). (D) DSC cerebral blood volume map. The hyperperfusion signal intensities correlate with those seen on cerebral blood flow map. (E) *f* map clearly highlights the area with high perfusion in the margins of the tumor (*arrows*), which is more evident than in C and D. (F) *D* map shows small restricted diffusion area (average *D* value = 0.895 x 10^−3^ mm^2^/s) predominantly in the anterior tumor margin (*arrows*). (G) D* map shows increased fast-diffusion values in the tumor tissue (arrows).

Table 2 shows the associations for the values of DWI parameters, DSC-MRI parameters, and IVIM-metrics and the correlations between them for CER and NCER. The IVIM-metric *f*_CER_ correlated moderately with rCBF_CER_ and rCBV_CER_ (*R* = 0.65;*P* = 0.01 and *R* = 0.49;*P* = 0.04, respectively) (Fig 1) and with *D*_CER_ and D*_CER_ (*R* = -0.68;*P* = 0.01 and *R* = 0.80;*P*<0.001, respectively). D*_CER_ also correlated with rCBF_CER_ (*R* = 0.71;*P*<0.001). rCBF_CER_ significantly correlated with rCBV_CER_ (*R* = 0.82;*P*<0.001). Moreover, *f*_CER_ correlated with D*_NCER_ (*R* = 0.85;*P* = 0.04) and with rCBF_NCER_ (*R* = 0.56;*P* = 0.03) and rCBV_NCER_ (*R* = 0.56;*P* = 0.03).

**Table 2:** Correlations between IVIM-metrics and DSC-MRI parameters for CER and NCER^a^.

Pearson r / *P*-value	A	B	C	D	E	F	G	H	I	J	K	L	M	N
**A *= f***_**CER**_**(%)**		**0.01**	**<0.01**	0.45	**0.01**	**0.04**	**<0.01**	0.18	0.31	0.62	**0.04**	0.72	**0.03**	**0.03**
**B *= D***_**CER**_**(x10**^**3**^**mm**^**2**^**/s)**	**-0.68**		0.09	0.11	0.73	0.63	**0.01**	0.41	0.08	0.58	0.67	0.58	0.91	0.25
C = D*_CER_**(x10**^**-3**^**mm**^**2**^**/s)**	**0.80**	-0.46		0.72	**<0.01**	0.12	**<0.01**	0.21	0.16	0.85	0.94	0.52	0.50	0.48
**D = ADC**_**CER**_**(x10**^**-3**^**mm**^**2**^**/s)**	-0.21	0.43	0.10		0.34	0.97	0.36	0.34	0.99	0.65	0.45	0.40	0.88	0.44
**E = rCBF**_**CER**_**(ml/100g/min)**	**0.65**	-0.10	0.71	-0.27		**<0.01**	0.42	**0.03**	0.97	0.99	0.06	0.85	0.05	0.43
**F = rCBV**_**CER**_ **(ml/100g)**	**0.49**	-0.14	0.42	-0.01	**0.82**		0.41	**0.02**	0.96	0.81	**0.04**	0.90	0.06	0.44
**G = max rCBF**_**CER**_**(ml/100g/min)**	**0.65**	**-0.61**	**0.71**	-0.25	0.22	0.23		0.32	0.08	0.95	0.60	0.89	0.10	0.82
**H = max rCBV**_**CER**_**(ml/100g)**	0.37	-0.23	0.34	-0.27	**0.57**	**0.61**	0.27		0.14	0.72	**0.03**	0.50	0.65	0.22
**I *= f***_**NCER**_**(%)**	0.31	0.47	-0.38	0.99	0.97	0.96	-0.47	0.4		0.11	0.06	0.67	0.66	0.75
**J *= D***_**NCER**_**(x10**^**-3**^**mm**^**2**^**/s)**	0.62	0.58	0.85	0.65	0.99	0.81	0.02	-0.1	0.11		0.93	0.07	0.12	0.59
K = D*_NCER_**(x10**^**-3**^**mm**^**2**^**/s)**	**0.85**	0.12	0.94	0.45	0.06	**0.53**	-0.15	**0.56**	0.06	-0.02		0.14	0.08	**0.01**
**L = ADC**_**NCER**_**(x10**^**-3**^**mm**^**2**^**/s)**	0.10	-0.16	0.18	0.4	0.85	0.9	0.04	0.19	0.12	0.47	0.40		0.12	0.05
**M = rCBF**_**NCER**_**(ml/100g/min)**	**0.56**	-0.03	0.19	-0.04	0.86	0.84	0.44	-0.13	-0.12	-0.42	-0.47	-0.41		**<0.01**
**N = rCBV**_**NCER**_**(ml/100g)**	**0.56**	0.32	-0.20	0.22	-0.22	0.44	0.06	-0.34	-0.09	-0.15	-0.65	-0.52	**0.77**	

^**a**^ Below the diagonal line are the correlation coefficients; above the diagonal are the P-values of the Pearson correlations. Significant correlations are highlighted in bold.

### Survival analysis

**Fig 2:**
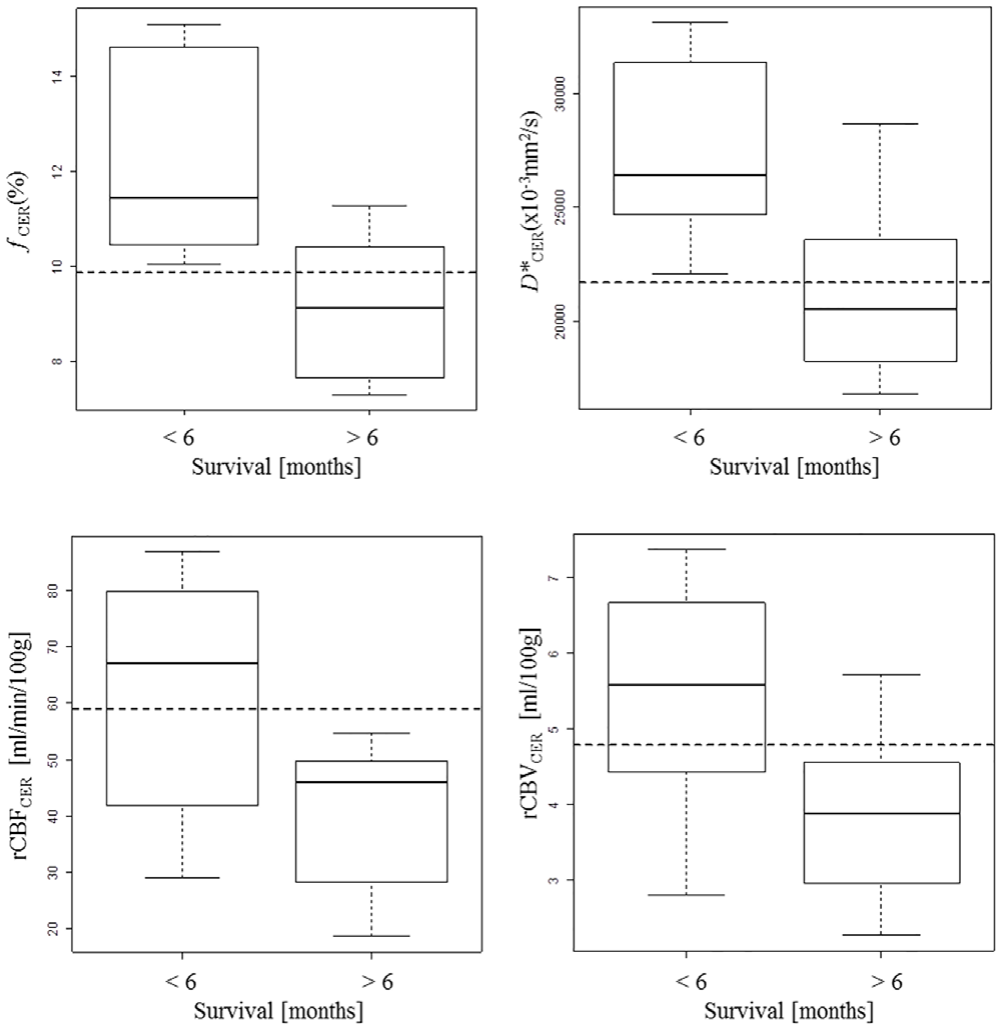
Boxplots of DSC-MRI parameters and IVIM-metrics for CER according 6-month survival.

**Fig 3:**
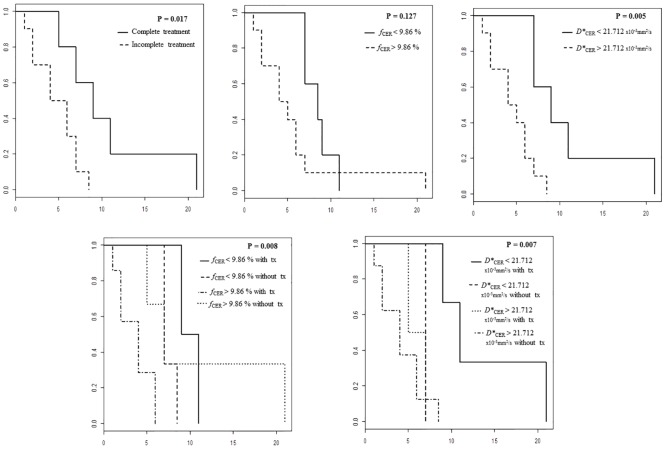
Kaplan-Meier survival curves comparing survival rates for treatment and for pre-specified cutoff values of *f*_CER_ and D*_CER_ (*upper row*) and for these cutoffs according to treatment received (*lower row*) Surgery, radiotherapy, and chemotherapy with concomitant and adjuvant temozolomide was considered standard treatment (tx).

Table 3 compares the IVIM-metrics and DSC-MRI parameters for patients who survived more than 6 months versus those who survived less than 6 months. Values for *f*_CER_, D*_CER_, rCBF_CER_, and rCBV_CER_ were significantly higher in patients who survived less than 6 months (Fig 2). The proportion of patients that received standard treatment was higher in the group that survived more than 6 months. Table 4 shows the cutoff values for *f*_CER_, D*_CER_, rCBF_CER_, and rCBV_CER_ for predicting survival. The cutoff *f*_CER_>9.86% had the highest AUC for predicting 6-month survival (100% sensitivity, 71.4% specificity, 100% positive predictive value (PPV), and 80% negative predictive value (NPV); AUC 0.893). The cutoff D*_CER_>21.712x10^−3^mm^2^/s yielded 100% sensitivity, 71.4% specificity, 80% PPV, and 100% NPV with an AUC of 0.857. The treatment was a significant predictor for 6-month survival (85.7% sensitivity, 57.1% specificity, 70% PPV and 80% NPV; AUC 0.723). In the Cox regression analysis, treatment was the most important factor (hazard ratio 5.484, 95% confidence interval 1.162–25.88, *P* = 0.031). In the multivariate analysis, only *f*_CER_ combined with treatment predicted survival 100% (Table 4). Survival rate was significantly shorter in patients with high values of *f*_CER_ (*P* = 0.008) and D*_CER_ (*P* = 0.007) independently of the treatment received (Fig 3).

**Table 3:** Clinical data, and diffusion and perfusion parameters in contrast-enhancing and non-enhancing regions according to survival^a^.

Characteristic	Survival < 6 months (n = 8)	Survival > 6 months (n = 7)	p-value
**Male/Female**	37.5% /62.5%	57.1% /42.9%	0.447
**Age**	68.5 (63.75–72.5)	69 (59.5–75)	1.00
**Motor deficit (%)**	50	42.9	0.782
**Language deficit (%)**	50	14.3	0.143
**Karnofsky Performance Score**	90 (80–100)	90 (85–95)	0.903
**CER (mL)**	16.05 (12.28–19.67)	13.82 (8.48–23.78)	0.779
**Necrosis (mL)**	5.47 (2.95–10.18)	4.11 (2.15–7.05)	0.397
**NCER (mL)**	49.66 (34.43–70.23)	33.99 (26.01–58.11)	0.281
***f***_**CER**_ **(%)**	11.43 (10.64–14.53)	9.13 (7.63–10.41)	**0.009**
***D***_**CER**_ **(x10**^**-3**^**mm**^**2**^**/s)**	0.997 (0.904–1.056)	1.115 (1.040–1.260)	0.121
D*_CER_ **(x10**^**-3**^**mm**^**2**^**/s)**	26.448 (24.774–30.702)	20.507 (18.254–23.601)	**0.021**
**ADC**_**CER**_**(x10**^**-3**^**mm**^**2**^**/s)**	100.39 (88.9–113.83)	123.51 (106.48–131.59)	0.072
**rCBF**_**CER**_ **(ml/100g/min)**	67 (47.12–78.48)	46.15 (28.24–49.87)	**0.04**
**rCBV**_**CER**_ **(ml/100g)**	5.57 (4.67–6.51)	3.87 (2.95–4.55)	**0.04**
**max rCBF**_**CER**_ **(ml/100g/min)**	165.9 (156.2–173.4)	140.5 (133.2–147.7)	0.07
**max rCBV**_**CER**_ **(ml/100g)**	14.1 (11.0–16.8)	10.6 (9.9–11.5)	**0.04**
***f***_**NCER**_ **(%)**	2.27 (1.18–3.11)	2 (1.94–3.15)	0.602
***D***_**NCER**_ **(x10**^**-3**^**mm**^**2**^**/s)**	1.381 (1.267–1.662)	1.486 (1.305–1.787)	0.779
D*_NCER_ **(x10**^**-3**^**mm**^**2**^**/s)**	6.352 (3.752–7.204)	3.764 (2.523–4.264)	0.094
**rCBF**_**NCER**_ **(ml/100g/min)**	23.69 (13.37–33.67)	22.92 (17.39–23.68)	0.779
**rCBV**_**NCER**_ **(ml/100g)**	1.06 (0.85–1.43)	1.75 (1.31–1.86)	0.336
**Treatment**			0.067
**Standard (n)**	1	4	
**Non-standard (n)**	7	3	
**Survival (months)**	4 (2–5.25)	8.5 (7–10)	**0.001**

^**a**^ Significant p-values are highlighted.

**Table 4:** Survival prediction: summary of class performance and hazard ratios for associations between imaging features and overall survival^a^.

Variable	ROC analysis						Cox regression model	
	Cutoff	AUC	Sensitivity	Specificity	PPV	NPV	Hazard ratio (95% CI)	p-value
*Univariate Analysis*								
***f***_**CER**_ **(%)**	9.860	0.893 (0.723–1.063)	1.000	0.714	1.000	0.800	1.193 (0.941–1.513)	0.145
D*_CER_ **(x10**^**-3**^**mm**^**2**^**/s)**	21.712	0.857 (0.648–1.067)	1.000	0.714	0.800	1.000	1.000 (1.000–1.000)	0.068
**rCBF**_**CER**_ **(ml/100g/min)**	59.010	0.821 (0.593–1.050)	0.625	1.000	1.000	0.700	1.025 (0.9934–1.057)	0.123
**rCBV**_**CER**_ **(ml/100g)**	4.780	0.821 (0.599–1.044)	0.750	0.857	0.857	0.750	1.158 (0.7698–1.742)	0.481
**max rCBF**_**CER**_ **(ml/100g/min)**	155.25	0.786 (0.533–1.000)	0.750	0.857	0.857	0.750	1.032 (0.995–1.069)	0.089
**max rCBV**_**CER**_ **(ml/100g)**	10.765	0.821 (0.598–1.000)	0.875	0.714	0.750	0.714	1.044 (0.864–1.261)	0.658
**Treatment**	1.500	0.723 (0.490–0.956)	0.857	0.571	0.700	0.800	5.484 (1.162–25.88)	0.031
*Multivariate Analysis*								
***f***_**CER**_ **and treatment**		1.000	1.000	1.000	1.000	1.000		
D*_CER_ **and treatment**		0.929	1.000	0.857	0.889	1.000		
**rCBF**_**CER**_ **and treatment**		0.929	0.875	0.857	0.875	0.857		
**rCBV**_**CER**_ **and treatment**		0.893	1.000	0.750	0.778	1.000		

^a^Data are hazard ratio estimates, with 95% confidence intervals in parentheses, for variables included in the Cox regression model (imaging features plus clinical variables) for the analysis of the association between the imaging features and overall survival after adjusting for standard clinical variables. Likelihood ratio test of this model versus the null model: P = 0.047 (test statistic = 15.66 with eight degrees of freedom).

### Intraobserver Reliability

Intraobserver agreements were almost perfect for DSC-MRI CER indexes (ICC = 0.916), DSC-MRI NCER indexes (ICC = 0.949), *f*_CER_ (ICC = 0.979), *f*_NCER_ (ICC = 0.983), *D*_CER_ (ICC = 0.930), and *D*_NCER_ (ICC = 0.98); intraobserver agreement was good for DSC gray matter indexes (ICC = 0.731). The Bland-Altman plots confirmed the high intraobserver reliability (S1 Fig).

## Discussion

Our preliminary results are an important step in collecting evidence about the feasibility and usefulness of IVIM imaging as a quantitative method to measure perfusion in glioblastoma. To our knowledge, this is the first study to demonstrate the usefulness of IVIM-metrics in predicting survival in patients with newly diagnosed glioblastoma. We found that patients with increased *f*_CER_ and D*_CER_ had significantly shorter survival independently of the treatment they received. Although a detailed analysis will require more data, the following can be deduced from this small cohort: the cutoffs *f*_CER_ = 9.86% and D*_CER_ = 21.712 x10^−3^mm^2^/s on pretreatment MRI yielded the highest predictive power for 6-month survival (AUC 0.893 and 0.857, respectively). However, in the Cox regression models, treatment was the only significant variable (P = 0.031), although D*_CER_ was nearly significant (*P* = 0.068).

The standard of care for newly diagnosed glioblastoma is now maximum safe surgical resection followed by radiotherapy plus concomitant and adjuvant chemotherapy with temozolomide [36]. This approach is based on a landmark phase III trial that reported median survival after surgery of 14.6 months in patients randomized to receive radiotherapy plus temozolomide compared to 12.1 months in those that receive radiotherapy alone [37], and other studies have corroborated this survival benefit [36,38]. Our results are consistent with these reports; importantly, however, we also found that adding f_CER_ data to treatment data enable survival to be predicted with an accuracy of 100%. Our preliminary results suggest that patients treated with the standard of care who had f_CER_ or D*_CER_ values over a pre-specified cutoff had worse survival than those who had f_CER_ or D*_CER_ values below the cutoff. Therefore, IVIM-metrics may help tailor the therapeutic approach in upcoming studies.

We found a negative correlation between *f*_CER_ and *D*_CER_, probably because regions with highest tumor cellularity almost certainly correspond to regions with highest vascularity. Bisdas et al. [12] revealed that IVIM fitting of the diffusion data allowed the contribution of perfusion to be separated from the contribution of true diffusion, thus providing better information than the apparent diffusion coefficient (ADC) for discriminating between low- and high-grade gliomas. Although one study found lower ADC values in high-grade gliomas than in lower-grade gliomas [39], another study reported considerable overlap in ADC values between low- and high-grade gliomas [40].

We found a strong positive correlation between *f*_CER_ and D*_CER_ and moderate positive correlations between *f*_CER_ and rCBF_CER_ and rCBV_CER_. The correlation with rCBF_CER_ was stronger than the correlation with rCBV_CER_ because rCBV is sensitive to vessel wall permeability, whereas *f* reflects only blood flowing in small vessels. Our results are in line with those recently reported by Federau et al. [11] in 21 gliomas (16 high-grade and 5 low-grade). They found that *f* correlated moderately with rCBV (r = 0.59) and, in the regions of maximum *f*, was significantly higher in the high-grade group.

In a recent study, Iima et al. [28] used a 17.2-T MR scanner to investigate the IVIM perfusion model and 2 non-Gaussian diffusion models for evaluating tissue characteristics in rodent gliomas [41,42]. IVIM maps highlighted tumor areas as generally heterogeneous, as confirmed by histology, and *f* was significantly higher in tumors than in contralateral tissue (P<0.001), as would be expected given neovascularization. Indeed, there was a significant positive correlation between *f* and microvessel density (R = 0.56, *P*<0.05), and a negative correlation was found between cellularity and *D* (R = -0.70, *P*<0.01).

Infiltrating tumor cells are present in the perivascular spaces in areas of vasogenic edema around the CER [43]. Higher rCBV ratios have been found in NCER surrounding gliomas than in the NCER surrounding metastatic lesions [44]. As would be expected given the vascularity of the CER and NCER, we found positive correlations between *f*_CER_ and rCBF_NCER_ and rCBV_NCER_. Furthermore, we found that *f*_CER_ strongly correlated with D*_NCER_. The D*value is considered proportional to mean capillary segment length and average blood velocity [18]; like the *f* value, the D*value may depend on the attenuation of the tumor microvessels and may correlate with the degree of angiogenesis with intact vessels, probably more frequent in the NCER, in terms of basement membrane thickness and pericyte coverage [45]. Further research could shed more light on potential IVIM-metrics to characterize the NCER of high-grade gliomas.

Several authors have used IVIM to separate the diffusion and perfusion components of DWI data, highlighting its potential value in clinical practice[12,18,46]. In healthy volunteers, Wirestam et al. [46] demonstrated modest but significant correlations between *f* and CBV (R = 0.56;*P*<0.001) and between CBF obtained from the median value of D* in IVIM and CBF obtained from DSC-MRI (R = 0.35;*P*<0.001). Federau et al. [18] demonstrated that *f*, D*, and fD* parameters change gradually under a hypercapnia and hyperoxygenation challenge in the brain. Bisdas et al. [14] recently reported that *f* was significantly correlated with DSC-derived vascular plasma volume and vascular transit time in healthy brain tissue, whereas in tumor regions, DSC-derived plasma flow was positively correlated with D* and inversely correlated with *f*.

The IVIM method has many theoretical advantages over DSC-MRI. Unlike DSC-MRI, IVIM perfusion-related parameters can be obtained using DWI datasets without the need for intravenous contrast agents, an important advantage considering that some agents are contraindicated in some patients due to the risk of nephrogenic systemic fibrosis [47]. Moreover, whereas DSC-MRI requires knowledge of the arterial input function, IVIM is intrinsically quantitative and the intravoxel excitation and readout obviates the need for this measurement [48,49]. Likewise, DSC-MRI requires a contralateral normalization measurement that can be difficult to obtain because of anatomical distortion, whereas IVIM-metrics are obtained through direct assessment of the tumor. IVIM diffusion and perfusion parameters might also be useful for guiding biopsy within gliomas [50]. Federau et al. [13] recently reported that T_2_-prepared IVIM inversion recovery acquisition seems to increase the quantitative blood volume contrast and contrast-to-noise ratio compared to standard IVIM acquisition and DSC-MRI, improving subjective lesion detection, contrast quality, and diagnostic confidence.

Some important limitations of this study merit comment. This pilot study was done at a single center, and the patient sample was too small to draw any definite conclusions about the usefulness of IVIM-metrics for patient management and survival prediction. The reported cutoffs most probably are not optimal and need to be validated. Although the large vessels try to be excluded, even small vessels could potentially affect the signal intensity or calculated IVIM map. IVIM is sensitive to motion if the curve is fitted on a voxel-by-voxel basis, so unavoidable patient movements may be problematic. Susceptibility inhomogeneities, as might occur around the petrous apex or the paranasal sinuses or due to the presence of blood postoperatively, for example, can harm the IVIM signal; however, they are also problematic in DSC-MRI. Analyzing the non-Gaussian diffusion behavior of water (kurtosis or biexponential model) can potentially provide information on microcirculation and tissue microstructure [28], but our diffusion images were acquired with maximum b-value of 1000 s/mm^2^, limiting our ability to go deeper in the analysis of non-Gaussian water movement due to cellular membrane boundaries [51,52]. The manual placement of the ROIs was subjective. Differences in slice thickness and spatial resolution in DSC, IVIM and T1WI would raise issues of systematic bias to match across different modalities, In our study, *f* and D* maps was particularly noisy and did not show clear anatomical findings (Fig 1), which might be expected since these parameters should be tissue specific (e.g. GM has much higher perfusion than WM). Better signal to noise ratio from higher field [14] or more averages must be sought for future studies. Partial-volume contamination from cerebrospinal fluid or necrotic areas may have varied during the study. IVIM and DSC have similar spatial resolution; however, DSC data probably has higher SNR for deriving perfusion parameters, whereas the SNR in IVIM can make it difficult to extract the perfusion component reliably. Moreover, IVIM suffers just as much as DSC from the effects of large-vessel partial volumes, but the IVIM equation is still an approximation of the signal model to the data and is not really quantitative in the sense that the ADC measurement can be (when data from low b-values are excluded). Another limitation of IVIM is that the level of perfusion must be high enough before IVIM can reliably detect and measure a perfusion-related parameter. Grech-Sollars et al [53] found that the IVIM parameter f had a poorer inter-scanner coefficient of variation when scanners of different field strengths were combined for normal, and the parameter was also affected by the scan acquisition resolution, for which perfusion is lower than the GBM angiogenic core.

## Conclusions

In summary, IVIM seems feasible for evaluating the diffusion and perfusion characteristics of glioblastoma, and *f*_CER_ and D*_CER_ correlate well with response to therapy and survival. Knowing which patients will respond better to treatment is important for individualizing care, so these parameters might help improve outcomes. Further studies are warranted to test the generalizability of our findings to other cohorts to determine whether IVIM-metrics can be used as perfusion biomarkers in gliomas.

## Supporting Information

S1 Fig Bland-Altman plots. The plots show that practically all the values are within the confidence limits.
(TIF)
