# Supplementary figures and images for: Intravoxel Incoherent Motion Metrics as Potential Biomarkers for Survival in Glioblastoma
Source: PLoS One. 2016 Jul 7;11(7):e0158887. doi: 10.1371/journal.pone.0158887 (PMC4936699; doi:10.1371/journal.pone.0158887)

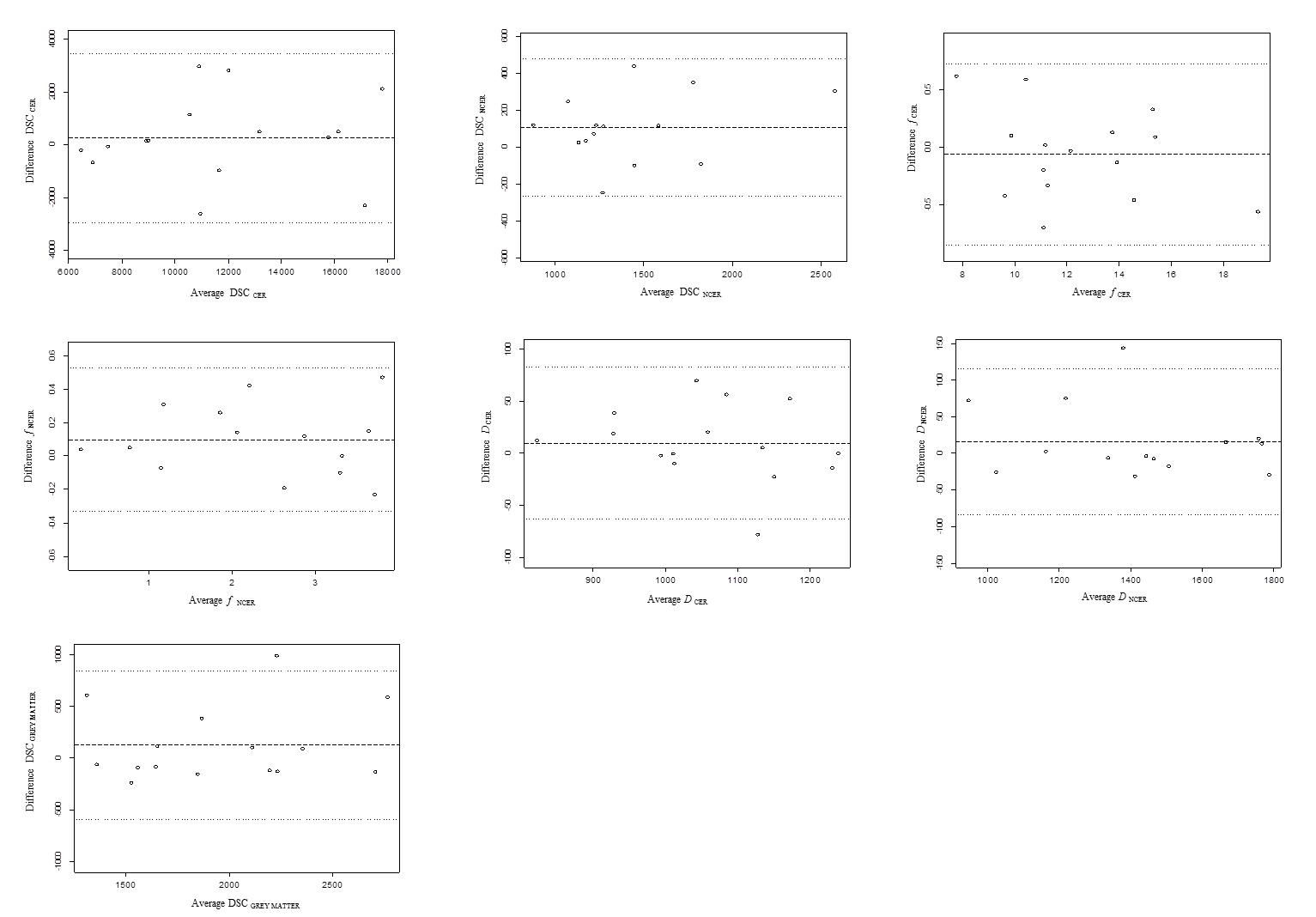

Supplement: S1 Fig — The plots show that practically all the values are within the confidence limits. (TIF) [file pone.0158887.s001.tif]
